# Presentation of SLE in UK primary care using the Clinical Practice Research Datalink

**DOI:** 10.1136/lupus-2016-000172

**Published:** 2017-02-10

**Authors:** Alison L Nightingale, Julie E Davidson, Charles T Molta, Hong J Kan, Neil J McHugh

**Affiliations:** 1 Department of Pharmacy & Pharmacology, University of Bath, Bath, UK; 2 Worldwide Epidemiology, GlaxoSmithKline R&D, Stockley Park, London, UK; 3 U.S. Health Outcomes, GlaxoSmithKline, Research Triangle Park, North Carolina, USA; 4 U.S. Medical Affairs, GlaxoSmithKline, Philadelphia, Pennsylvania, USA; 5 Royal National Hospital for Rheumatic Diseases, Bath, UK

**Keywords:** Systemic Lupus Erythematosus, Primary Care, Epidemiology, Clinical Practice Research Datalink, Diagnostic Delay

## Abstract

**Objectives:**

To describe the presenting symptoms of SLE in primary care using the Clinical Practice Research Database (CPRD) and to calculate the time from symptom presentation to SLE diagnosis.

**Methods:**

Incident cases of SLE were identified from the CPRD between 2000 and 2012. Presenting symptoms were identified from the medical records of cases in the 5 years before diagnosis and grouped using the British Isles Lupus Activity Group (BILAG) symptom domains. The time from the accumulation of one, two and three BILAG domains to SLE diagnosis was investigated, stratified by age at diagnosis (<30, 30–49 and ≥50 years).

**Results:**

We identified 1426 incident cases (170 males and 1256 females) of SLE. The most frequently recorded symptoms and signs prior to diagnosis were musculoskeletal, mucocutaneous and neurological. The median time from first musculoskeletal symptom to SLE diagnosis was 26.4 months (IQR 9.3–43.6). There was a significant difference in the time to diagnosis (log rank p<0.01) when stratified by age and disease severity at baseline, with younger patients <30 years and those with severe disease having the shortest times and patients aged ≥50 years and those with mild disease having the longest (6.4 years (IQR 5.8–6.8)).

**Conclusions:**

The time from symptom onset to SLE diagnosis is long, especially in older patients. SLE should be considered in patients presenting with flaring or chronic musculoskeletal, mucocutaneous and neurological symptoms.

## Introduction

The time from symptom onset to diagnosis of SLE has been reported to be approximately 2 years^1–3^ and increasing awareness of SLE has reduced this time from symptom report to a physician to diagnosis over the past 30 years.^2^ It has been reported that children, males and patients with late-onset SLE (over the age of 50) have a longer time from first symptom to diagnosis than adult-onset SLE,^1–3^ possibly due to the higher diagnostic suspicion of SLE in women of reproductive age.^3^ There is evidence to suggest that damage can occur during the early years of the disease and that this is related to age at diagnosis and disease duration^4^ and that a decrease in diagnostic delay contributes to improved survival and quality of life.^2^

In the majority of patients, constitutional (especially fatigue), cutaneous and musculoskeletal symptoms are the first manifestations of SLE.^5–10^ Children tend to have a more severe onset of symptoms^11–13^ with haematological, neurological and renal involvement occurring more commonly than in adult-onset SLE.^7^ Patients with late-onset SLE (≥50 years) tend to have a more insidious onset of disease with severe manifestations being infrequent;^14–18^ however, they are also more likely to have greater damage at diagnosis, a higher frequency of comorbidities and a higher risk of premature mortality than those with an earlier onset of SLE.^9^ ^18–21^

To date, studies reporting time from symptom onset to diagnosis of SLE have been conducted in specialist secondary care settings using patient interviews or medical record review. Accurate recall of the timing of symptom onset will depend on how long ago the symptoms manifested and whether or not patients attribute specific symptoms to their eventual diagnosis. There have been no studies reporting on the prospective evolution of symptoms of SLE using data collected in primary care. The aim of the study was to describe the presenting symptoms and signs leading up to a diagnosis of SLE, using data prospectively collected in primary care and to calculate the time from the first record of joint or skin symptoms in the 5 years before diagnosis to SLE diagnosis, stratified by age, sex and disease severity at diagnosis.

## Methods

### Data source

The Clinical Practice Research Datalink (CPRD, formerly the General Practice Research Database (GPRD)) is, to our knowledge, the world's largest database of longitudinal primary care records. It contains the anonymised primary care records for approximately 8.4% of the UK population of which it is generally representative in terms of age and sex structure.^22^ Patients enter the CPRD on the latest of (a) their date of birth, (b) the date that they register with their general practitioner (GP) or (c) the date on which their GP practice starts to contribute data to the CPRD. This is their ‘left censoring’ date. They leave the CPRD on the earliest of (a) their date of death, (b) the date they leave their GP practice or (c) the date that their GP stops contributing data to the CPRD. This is their ‘right censoring’ date. Diagnoses are entered using Read codes, prescription data are generated when GPs issue prescriptions via their office computer. Laboratory test data are available but for the majority of the study period were limited to tests conducted in primary care. The CPRD has been previously used to describe the epidemiology of SLE in the UK.^23–27^

### Case identification

The study period ran from 1 January 2000 to 31 December 2012. The study population included all permanently registered patients contributing research standard data to the CPRD during the study period. There were no age restrictions to the study population. We searched the study population using Read codes for SLE and lupus erythematosus (LE) to identify incident cases of SLE. Incident cases of SLE were defined as those with a first diagnosis of SLE or LE between 1 January 2000 and 31 January 2012 and who had at least 2 years of data between their left censoring date and first date of SLE diagnosis. We excluded patients who had Read codes for drug-induced and those with Read codes indicating isolated cutaneous lupus. The date of SLE diagnosis was defined as the earliest of: (a) the first record of SLE diagnosis or (b) the first record of a prescription for an immunosuppressant or hydroxychloroquine without an alternative explanatory diagnosis.

**Figure 1:**
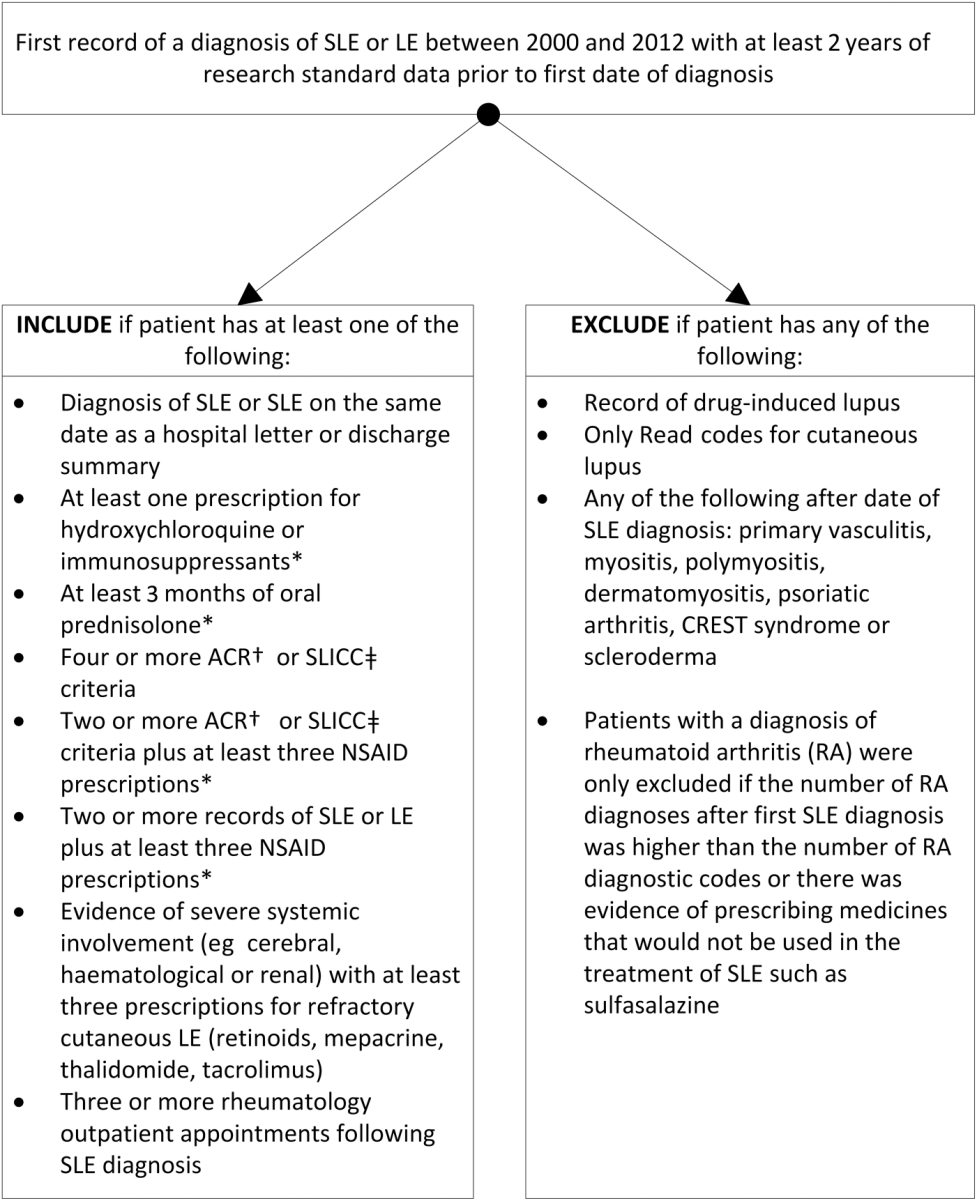
Algorithm for the identification of patients with a diagnostic code for SLE. *Without an alternative explanatory diagnosis. †ACR criteria.^28^ ^29^ ‡SLICC criteria.^43^ CREST, limited cutaneous systemic sclerosis; LE, lupus erythematosus; ACR, American College of Rheumatology; SLICC, Systemic Lupus International Collaborating Clinics; NSAID, non-steroidal anti-inflammatory drugs.

SLE is usually classified, for the purposes of research, using the American College of Rheumatology (ACR) classification criteria for SLE.^28^ ^29^ The CPRD is a primary care database and data are entered by GPs for the purposes of the clinical management of their patients, not specifically for research. This means that many of ACR criteria would not be routinely recorded on the CPRD. For this reason, we searched for supporting evidence of SLE diagnosis in order to classify patients with a diagnostic code for SLE as cases of SLE and to thereby exclude patients with a diagnosis of LE who were more likely to have only cutaneous lupus than SLE. Figure 1 shows the algorithm for the identification of cases of SLE that was based on our previous study of the epidemiology of SLE using the GPRD.^23–25^

### Coding of presenting symptoms and signs of SLE

**Figure 2:**
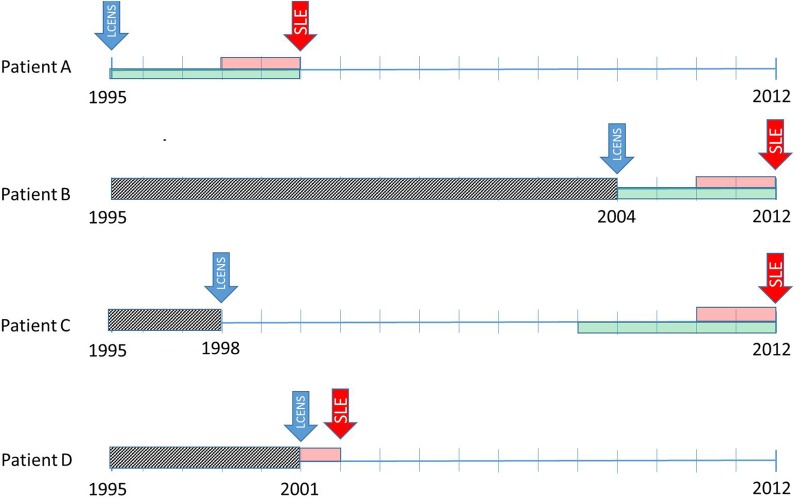
Timelines used to identify symptoms recorded in the 5 years before SLE diagnosis taking into account data censoring. *Patient A*. Joins the Clinical Practice Research Database (CPRD) in 1995 (their left censoring date (LCENS—the blue arrow) and is diagnosed with SLE in 2000 (the red arrow). We require at least 2 years between their LCENS date and their date of SLE diagnosis to include them as an incident case of SLE (the pink box). We code symptoms of SLE in the 5 years before SLE diagnosis (the green box) and we can use all of those 5 years since their LCENS date is in 1995. *Patient B*. Joins the CPRD in 2004 (their LCENS date) therefore, we cannot see their medical records before 2004 (the grey box). They have at least 2 years of data between their LCENS (2004) and date of SLE diagnosis (2012). We can only use the 4 years of data between their date of SLE diagnosis and LCENS date to look for symptoms of SLE. *Patient C*. Joins the CPRD in 1998 (their LCENS date). They have their diagnosis of SLE in 2012. For this patient, we have 14 years of research standard data (between their LCENS and date of diagnosis). Therefore, they are an incident case of SLE as they have at least 2 years of data before their SLE diagnosis. We can use symptoms recorded in the 5 years before diagnosis to investigate the onset of SLE. Although we have much more data, we decided not to use data more than 5 years before diagnosis because the data became very unstable due to low numbers. *Patient D*. Joins the CPRD in 2001 (LCENS date) and has their date of SLE diagnosis in 2002. They do not have 2 years of research standard data in their record between their LCENS date and date of SLE diagnosis and therefore would have been excluded from the study because we do not have sufficient data to ascertain whether the patient is an incident or prevalent case of SLE.

The medical record of each patient was searched for all Read coded diagnoses within the British Isles Lupus Activity Group (BILAG) domains^30^ that might have been associated with the onset of SLE in the 5 years before SLE diagnosis or to the patient's left censoring date, whichever was the earliest. Figure 2 shows the timelines for coding symptoms of SLE taking into account data censoring in the CPRD. Read code lists were developed by ALN, reviewed by a consultant rheumatologist (NMcH) and grouped according to the BILAG domains. In order to further investigate the proportion of patients with renal disease presenting before diagnosis, we split the BILAG renal domain into renal disease and treated hypertension. Treated hypertension was defined as (a) a diagnosis of hypertension plus at least one prescription for any antihypertensive medicine after diagnosis of hypertension or (b) the presence of any record of a hypertensive blood pressure reading plus at least one prescription for any antihypertensive medicine after the reading.

### Coding of disease severity at diagnosis

We classified patients as having mild-to-moderate or severe disease using prescribing data within the first 12 months after diagnosis. This method of classification was based on the ACR and European League Against Rheumatism treatment guidelines.^31^ ^32^ Patients with at least one prescription for an immunosuppressant or oral prednisolone at doses >7.5 mg/day for at least 30 days were classified as having severe disease at baseline. All other patients were classified as having mild-to-moderate disease. The case identification algorithm and coding of disease severity at diagnosis was based primarily on prescribing that is recorded in the CPRD. In the UK, the vast majority of prescribing that is hospital initiated is continued by GPs after the initial prescription and this continues under the guidance of the hospital specialists. While the first prescription for medicines, especially immunosuppressants, is initiated in a hospital setting, under shared-care arrangements, ongoing prescribing is generally the responsibility of the GP with the exception of biologics.^33^ ^34^ Shared-care arrangements in the UK ensure that there is a true transfer of information between secondary and primary care clinicians, therefore, the CPRD represents an almost complete record of prescribing for the majority of patients. It is rare that patients are prescribed biologics as a first-line therapy for SLE in the UK, therefore, prescribing that is recorded on the CPRD within the first 12 months after diagnosis should be reflective of disease severity at time of diagnosis.

### Statistical analyses

The proportion of patients with a current or historic record of any of the signs or symptoms within each BILAG domain^30^ was calculated for each 12-month period prior to diagnosis. The proportion of patients consulting their GP for any reason was calculated for each 12-month period prior to diagnosis. The denominator for each period was the number of SLE cases contributing data to the CPRD during that period taking left truncation of data into account.

Kaplan-Meier failure curves were constructed from the date of each patient's first record of a musculoskeletal symptom and of a mucocutaneous symptom in the 5 years before diagnosis to date of SLE diagnosis stratified by age, sex and disease severity at baseline and taking left data truncation into account. Log rank tests were used to test the equality of the failure functions. The median time from first musculoskeletal symptom and first mucocutaneous symptom SLE diagnosis was calculated using the failure data and stratified by severity of disease at baseline and 10-year age groups.

### Ethical approval

Ethical approval for the study was granted by the Independent Scientific Advisory Committee to the CPRD, protocol number 13_051.

## Results

The study population consisted of 9 651 514 eligible patients. There were 2303 patients with a first record of SLE diagnosis during the study period. Of these, 86 had an alternative diagnosis after their SLE diagnosis and 295 were not incident cases when their prescribing was taken into account and were therefore excluded. Of the remaining 1922 cases, 496 did not fulfil the inclusion criteria. Therefore, we included 1426 incident cases of SLE in the study (170 males (11.9%) and 1256 females (88.1%)); 1070 (75.0%) were classified as having mild-to-moderate disease at baseline and 356 (25.0%) were classified as having severe disease at baseline. As expected, very few patients (n=84, 5.9%) could be classified retrospectively as fulfilling four or more of the ACR criteria for SLE^28^ ^29^ and this was largely affected by a lack of comprehensive test data in the patient record. For example, there were 941 patients who had at least one ANA test in their CPRD record with a total of 6437 ANA tests recorded at any time before or after SLE diagnosis. Of these, 5596 tests (86.9%) did not have a test result recorded, 708 (11%) were recorded as ‘positive’, ‘high’ or ‘abnormal’ and 133 (2.0%) were recorded as ‘negative’ or ‘normal’.

The sex-specific incidence rates of SLE in the CPRD population during the study period were 0.7/100 000/year (95% CI 0.6 to 0.9) in males and 5.4/100 000/year (95% CI 5.1 to 5.8) in females. The mean age at SLE diagnosis was 49.4 years (SD 18.9, range 3–84 years) in the males and 47.7 years (SD 17.2, range 5–91 years) in the females. The mean duration of data contribution to the CPRD by patients prior to SLE diagnosis was 8.6 years (SD 4.8) and 1023 patients (71.7%) had at least 5 years of data prior to SLE diagnosis. Table 1 shows the characteristics of the included cases stratified by disease severity at diagnosis. Ethnicity was recorded in 658 of the cases (46.1%) and the majority of these were White (n=589, 89.5%).

**Table 1:** Characteristics of included incident cases of SLE, identified from the CPRD between 1 January 2000 and 31 December 2012, stratified by disease severity at diagnosis

	Disease severity at diagnosis		
	Mild-to-moderate (n=1070)	Severe (n=356)	All (n=1426)
Patient characteristics			
Males, n (%)	932 (74.2)	324 (25.8)	1256 (88.1)
Females, n (%)	105 (61.8)	65 (38.2)	170 (11.9)
Mean age at SLE diagnosis (years, SD)	48.3 (17.0)	46.6 (18.7)	47.9 (17.4)
Age at diagnosis, n (%)			
0–9 years	5 (0.5)	2 (0.6)	7 (0.5)
10–19 years	45 (4.2)	40 (11.2)	85 (6.0)
20–29 years	92 (8.6)	28 (7.9)	120 (8.4)
30–39 years	190 (17.8)	60 (16.9)	250 (17.5)
40–49 years	251 (23.5)	58 (16.3)	309 (21.7)
50–59 years	200 (18.7)	70 (19.7)	270 (18.9)
60–69 years	144 (13.5)	51 (14.3)	195 (13.7)
70–79 years	116 (10.8)	42 (11.8)	158 (11.1)
≥80 years	27 (2.5)	5 (1.4)	32 (2.2)
Supporting evidence of SLE diagnosis, n (%)			
Evidence of hospital treatment for SLE	624 (58.3)	231 (64.9)	855 (60.0)
Antimalarial prescribing after diagnosis	790 (72.5)	242 (68.0)	1032 (72.4)
Oral prednisolone (minimum 3 months) after diagnosis	257 (24.0)	318 (89.3)	575 (40.3)
Immunosuppressant prescribing after diagnosis	127 (11.9)	227 (63.8)	354 (24.8)
NSAID prescribing (minimum three prescriptions)	609 (56.9)	177 (49.7)	786 (55.1)
Therapy for refractory cutaneous LE after diagnosis	63 (5.9)	20 (5.6)	83 (5.8)
Evidence of severe systemic involvement at any time after diagnosis	13 (1.2)	22 (6.2)	35 (2.5)
Four or more ACR criteria^28^ ^29^	56 (5.2)	28 (7.9)	84 (5.9)
Four or more SLICC criteria^43^	73 (6.8)	43 (12.1)	116 (8.1)

Disease severity at baseline is defined as severe if the patient had at least one prescription for an immunosuppressant or a prescription for at least 30 days of treatment with oral prednisolone at a dose of 7.5 mg/day or more within 12 months of date of SLE diagnosis. All remaining patients were classified as having mild-to-moderate disease.

ACR, American College of Rheumatology; CPRD, Clinical Practice Research Database; LE, lupus erythematosus; NSAID, non-steroidal anti-inflammatory drugs; SLICC, Systemic Lupus International Collaborating Clinics.

**Figure 3:**
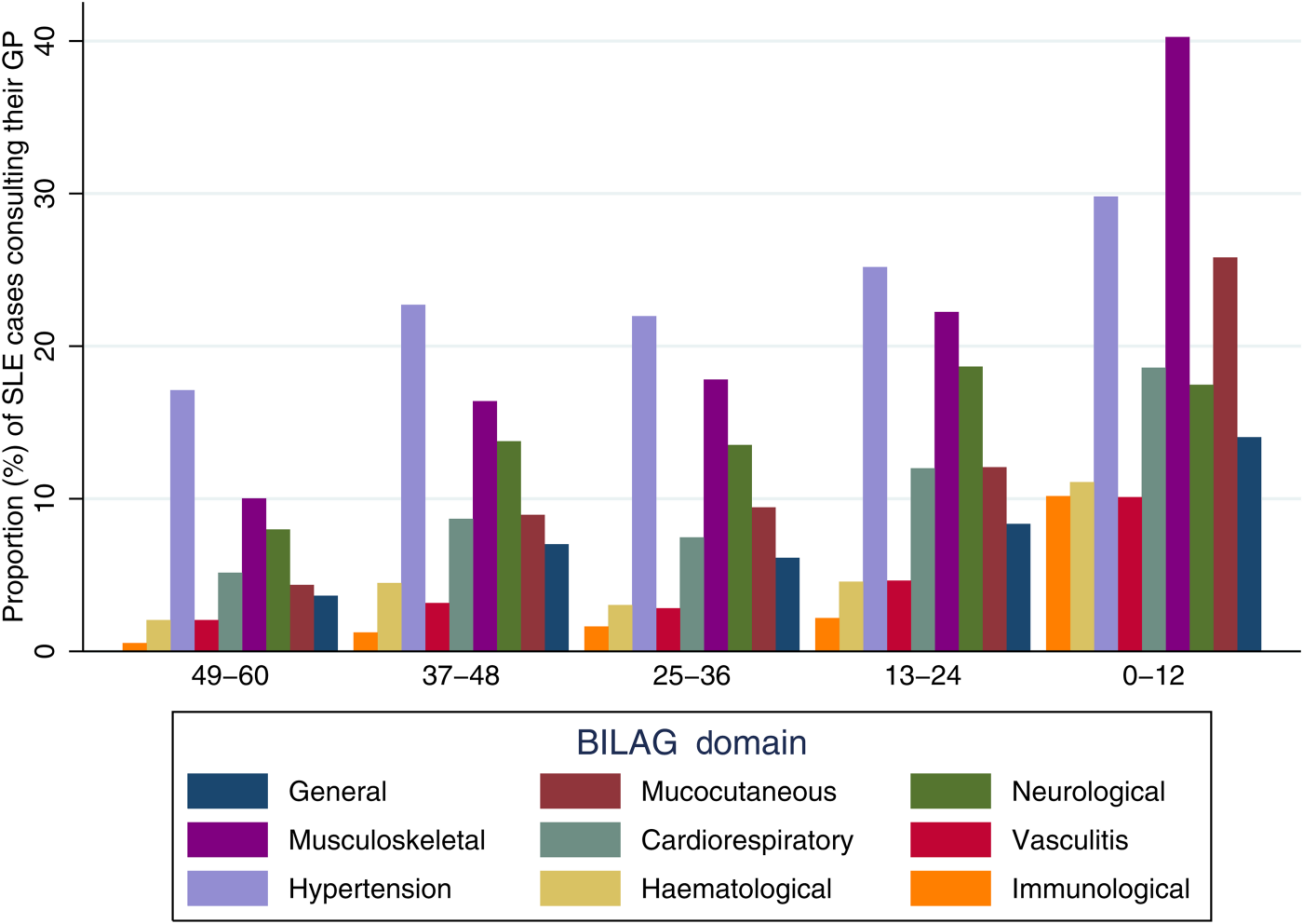
Proportion of patients consulting their general practitioner for symptoms within each British Isles Lupus Activity Group (BILAG) domain in the 5 years before SLE diagnosis (date of diagnosis between 1 January 2000 and 31 December 2012) by months before SLE diagnosis taking left data truncation into account in the denominator calculations for the 25–36, 37–48 and 49–60 months before diagnosis.

The proportion (%) of patients with a record of GP consultation for symptoms or signs within each BILAG^30^ domain during each time period before SLE diagnosis is shown in figure 3. The largest increases were in consultations for symptoms within the musculoskeletal, mucocutaneous and general domains. The proportion of patients having symptoms, signs or diagnoses in multiple domains also increased in the 5 years leading up to SLE diagnosis. The proportion of patients with three or more domains in their record increased from 18.7% (n=146) 5 years before diagnosis to 22.6% (n=205) 4 years before diagnosis, 321% (n=321) 3 years before diagnosis, 24.2% (n=231) 2 years before diagnosis and to 39.7% (n=458) in the year before SLE diagnosis. The median number of GP consultations for any reason increased in the 5 years before diagnosis from 1 (IQR 0–17) 54–48 months before diagnosis to 23 (IQR 11–43) in the 24–12 months before diagnosis and 38 (IQR 23–61) in the 0–12 months before diagnosis.

Within each BILAG domain, the most frequently recorded diagnoses were fatigue and malaise in 239 (16.8%). In the mucocutaneous domain, 43 patients had a record of a maculopapular eruption and 59 had a record of discoid rash with 426 patients (29.9%) having a record of ‘rash’ without any further details in the coded record. Symptoms in the musculoskeletal domain were the most commonly recorded in the 5 years before SLE diagnosis with a record of arthritis or arthralgia (n=836, 58.6%) and myalgia (n=126, 8.8%). Depression was the most commonly recorded diagnosis in the neurological domain (n=295, 20.6%). Very few patients had a record of psychosis (n=6, 0.04%)) or seizures (n=44, 3.1%)) in the 5 years before diagnosis. In the cardiorespiratory domain, chest pain (n=284, 19.9%) and dysponea (n=238, 16.7%) were the most commonly recorded diagnoses. One hundred and six patients (7.4%) had a record of serositis before SLE diagnosis. Within the vasculitis domain, the most commonly recorded symptoms or signs were thromboembolism (n=97, 6.8%) and Raynaud's phenomenon (n=81, 5.7%). The majority of patients with symptoms or signs within the renal domain had a record of treated hypertension (n=482, 33.8%), in the 5 years before diagnosis. Only 61 patients (4.3%) had a record of nephritis, proteinuria or cellular casts.

**Figure 4:**
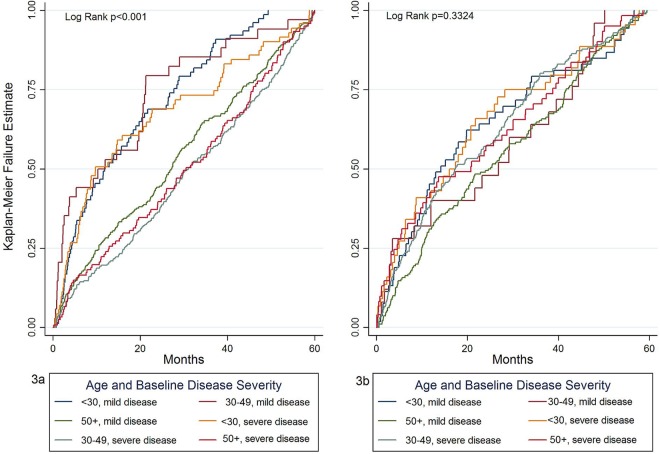
Kaplan-Meier failure estimate plots for the probability of diagnosis following a first record of a musculoskeletal symptom (3a) and first mucocutaneous symptom (3b) in the 5 years before diagnosis to date of SLE diagnosis, stratified by age at SLE diagnosis (<30, 30–49 and ≥50 years) and baseline disease severity (mild-to-moderate or severe) taking left data truncation into account. Disease severity at baseline is defined as severe if the patient has at least one prescription for an immunosuppressant or a prescription for at least 30 days of treatment with oral prednisolone at a dose of 7.5 mg/day or more within 12 months of date of SLE diagnosis with all other patients classified as having mild/moderate disease at baseline.

We investigated whether there were any specific combinations of symptoms before SLE diagnosis by tabulating frequencies of symptom combinations in the study population but no specific pattern of symptom combinations could be identified. Musculoskeletal or mucocutaneous symptoms or signs were recorded in 884 (62.0%) and 585 (41.0%), respectively, of patients in the 5 years before SLE diagnosis, therefore we constructed Kaplan-Meier failure curves from date of first musculoskeletal symptom and first mucocutaneous symptom record in the 5 years before date of diagnosis to SLE diagnosis stratified by a combination of age at SLE diagnosis (<30, 30–50 and >50 years) and baseline disease severity (mild-to-moderate or severe) (figure 4). There was significant inequality of the failure functions for musculoskeletal symptoms (log rank p<0.01) but not for mucocutaneous symptoms (log rank p=0.33). Patients initially presenting to their GP with musculoskeletal symptoms who were aged 30–49 years at SLE diagnosis and with mild disease at baseline and those aged 50 years or over at diagnosis irrespective of disease severity had a longer time from symptom presentation to SLE diagnosis than younger patients and particularly those with severe disease at diagnosis. There was no difference in the failure functions when the data were stratified for sex either for musculoskeletal symptoms (log rank p=0.62) or for mucocutaneous symptoms (log rank p=0.47). Table 2 shows the median times from first mucocutaneous and musculoskeletal symptom in the 5 years before diagnosis to date of diagnosis, stratified by age and baseline disease severity. In general, time from first musculoskeletal symptom to SLE diagnosis increased with increasing age and was lower for those with severe disease at baseline. There was less variation in time from first mucocutaneous symptom to SLE diagnosis when the data were stratified by age and baseline disease severity, consistent with the log rank test result from the Kaplan-Meier failure curves for mucocutaneous symptoms.

**Table 2:** Median time (months (IQR)) from first record of musculoskeletal and mucocutaneous symptom to date of SLE diagnosis on the CPRD stratified by age at diagnosis and baseline disease severity

	Mild-to-moderate disease		Severe disease	
Age group	n (%*)	Median months (IQR)	n (%*)	Median months (IQR)
Time from first musculoskeletal symptom to SLE diagnosis				
0–9 years	1 (0.07)	–	0	–
10–19 years	20 (1.4)	6.0 (2.8–27.7)	18 (1.3)	29.7 (2.3–21.3)
20–19 years	57 (4.0)	13.9 (5.1–26.5)	15 (1.1)	3.8 (1.2–20.6)
30–39 years	122 (8.6)	22.2 (7.9–39.9)	42 (2.9)	14.5 (5.7–37.8)
40–49 years	154 (10.8)	28.7 (14.6–46.0)	29 (2.0)	7.9 (3.8–29.2)
50–59 years	133 (9.3)	29.1 (17.5–16.2)	45 (3.2)	25.6 (10.6–37.3)
60–69 years	83 (5.8)	36.1 (15.8–49.6)	41 (2.9)	34.4 (14.4–45.4)
70–79 years	68 (4.8)	30.6 (16.9–49.4)	31 (2.2)	38.9 (12.7–48.0)
80+ years	21 (1.5)	33.9 (18.6–50.4)	4 (0.3)	44.3 (28.3–49.5)
Time from first mucocutaneous symptom to SLE diagnosis				
0–9 years	4 (0.3)	7.8 (3.9–12.7)	1 (0.07)	–
10–19 years	15 (1.1)	6.5 (1.7–26.1)	15 (1.1)	23.2 (2.9–45.4)
20–19 years	34 (2.4)	17.0 (9.5–45.1)	9 (0.6)	26.8 (3.5–43.1)
30–39 years	74 (5.2)	25.5 (10.2–41.2)	24 (1.7)	18.9 (6.4–28.2)
40–49 years	116 (8.1)	21.4 (9.9–41.7)	20 (1.4%)	14.3 (2.3–21.8)
50–59 years	65 (4.6)	13.5 (5.2–32.4)	22 (1.5)	20.9 (5.5–38.7)
60–69 years	77 (5.4)	17.5 (8.7–36.7)	20 (1.4)	23.7 (3.8–37.4)
70–79 years	53 (3.7)	23.0 (8.1–34.1)	16 (1.1)	9.7 (3.2–30.1)
80+ years	17 (1.2)	16.1 (7.2–28.6)	3 (0.2)	4.7 (2.8–41.6)

*Percentages calculated for all SLE cases (n=1426).

CPRD, Clinical Practice Research Database.

## Discussion

To our knowledge, this is the first study of the presentation of SLE that has used prospectively collected data on SLE symptom presentation prior to diagnosis from the UK primary care. While studies of the presentation of SLE conducted in specialist secondary care cohorts have the advantage of being able to establish the timing of symptoms that have not been reported to a patient's GP and determine whether symptoms and signs are likely to be related to their eventual diagnosis of SLE, the use of prospectively collected primary care data adds to the secondary care data in being able to describe how and when symptoms are reported by patients to their GPs, thereby getting a fuller picture of the complexity of the onset of symptoms of SLE. The data within the CPRD are collected during the course of routine clinical primary care and, as such, can be used to study the presentation of symptoms of SLE in a real-world environment. It is within this environment that the majority of patients with SLE will initially present therefore having an understanding of what these patients might look like to their GPs is essential in fully understanding barriers to diagnosis of SLE.

We found that musculoskeletal, mucocutaneous and neurological symptoms were the most commonly recorded symptoms prior to diagnosis and that there was a long lag between the onset of symptoms of SLE to diagnosis, particularly in patients with late-onset SLE (diagnosed at age 50 or older). We did not identify any specific symptom combinations confirming the insidious and varied nature of SLE presentation. However, we did note that the number of domains within which symptoms were being recorded increased towards the date of diagnosis suggesting that as patients become increasingly unwell and become multisystemic they consult their GP more frequently, leading to an eventual diagnosis of SLE. The proportion of patients consulting for musculoskeletal and mucocutaneous symptoms increased sharply in the 6 months before diagnosis with a corresponding rise in records for immunological abnormalities as a result of ANA testing. This suggests that GPs are mostly likely to suspect SLE in patients with these symptoms and that they are likely to be responsible for triggering the diagnostic process. However, we recognise that these are also symptoms of many, more common, disorders and without a general population control group, it is not possible to draw conclusions about the predictive value of these symptoms for an eventual diagnosis of SLE. Importantly, the presence of some symptoms that are used for the classification of SLE^28^ ^29^ such as photosensitivity, serositis, seizures, psychosis and renal disease are very rare in primary care prior to diagnosis. The finding that approximately a quarter of patients with SLE were being treated for hypertension is consistent with estimates of the general population prevalence of hypertension in men and women aged 35–74 years.^35^

The mean age of diagnosis of SLE in patients contributing data to the CPRD (49.4 years) is older than previously reported,^3^ ^13^ but consistent with previous studies using the GPRD.^23^ ^26^ The reason for this is not clear. It may be as a result of delayed recording of the diagnosis of SLE; however, this was accounted for by backdating the date of SLE diagnosis to the first prescription for medicines used for the treatment of SLE. In the UK, the majority of hospitals have a shared-care agreement with primary care where rheumatologists will retain responsibility for the management of their patient but GPs are usually responsible for issuing prescriptions under the guidance of the rheumatologist.^36^ Alternatively, this may reflect a higher burden of older-onset SLE in the UK than previously recognised with a milder presentation^18^ ^19^ perhaps meaning that these patients are not enrolled into secondary or tertiary care cohorts. This would also explain the low incidence of symptoms of severe SLE in this population.^9^ ^12^ Additionally, while ethnicity is not systematically recorded for all patients in the CPRD, the majority of those with a record of their ethnicity were White. In studies reporting the epidemiology of SLE in different world populations, it has been noted that age of onset tends to be older for White compared with Black patients.^37^ ^38^ Therefore, the older age at diagnosis of SLE in the CPRD population is likely to be affected by ethnicity.

The finding that musculoskeletal and mucocutaneous symptoms are the most frequently experienced first symptoms of SLE has been reported previously.^8^ ^9^ ^13^ These symptoms are not uncommon in the general population, which may explain the time from symptom report to diagnosis because unless the symptoms put together with others are put into the context of the suspicion of a relatively rare condition such as SLE, further investigations may not be pursued. However, the data suggest that once patients become multisystemic and are regularly presenting to their GP with symptoms that they do then receive a diagnosis.

The time from first symptom to diagnosis has been estimated to be between 0.5 and approximately 4 years.^3^ ^8^ ^9^ ^39–41^ The estimation of time from disease onset to diagnosis has been difficult to calculate in previous studies because it depends on the symptoms that the physician and patient attribute to SLE, the time between diagnosis and participating in a study of SLE onset and the accuracy of recall of the timing of symptoms that may have arisen many years previously. In this study, we aimed to describe the evolution of symptoms of SLE in primary care and found that symptoms of SLE were present in the medical records of patients for many years before their diagnosis. Ideally, we might have estimated time from symptom presentation to referral rather than SLE diagnosis, and recording of that diagnosis on the CPRD. However, referral is not consistently recorded in the CPRD, making using referral as an analysis end point of limited value. It was not possible to determine whether symptoms such as arthralgia, rash, headache, fatigue and depression were directly attributable to SLE or to other, more common, causes such as viral illness or injury. Nevertheless, the finding that symptoms of SLE are frequent in patients for years before their diagnosis, especially in older patients with SLE, should not be overlooked.

While the use of the CPRD has allowed us to investigate the evolution of symptoms of SLE in the UK primary care, these data source does have a number of limitations. First, no validation studies on algorithms used to identify SLE from the CPRD have been conducted. Given the nature of these algorithms, however, we would expect that it is more likely that patients at the extremities of the disease spectrum are the most likely to have been missed. Second, GPs contributing to the CPRD are only required to record symptoms and diagnoses that are clinically relevant or result in a new diagnosis or hospital referral rather than all symptoms that a patient reports. This issue is further compounded by the use of non-specific Read codes such as those for ‘rash’ or ‘arthralgia’. These coding practices and missing test data in the CPRD made it unfeasible to use the ACR classification criteria for SLE as an inclusion criteria to the study population and only 5.7% of patients had sufficient data in their record to fulfil four or more of the criteria. However, this is a limitation of data recording rather than an issue that should negate the findings of the study. The future potential for data linkage to the CPRD may present opportunities to extend the current study to further investigate whether earlier diagnosis may be possible in the future. Additionally, with laboratories increasingly entering laboratory test results into the primary care record of patients, over time the completeness of laboratory test results will increase in electronic databases such as the CPRD.

The potential for ‘flagging’ software could be explored in the future to enable GPs to more easily identify patterns of symptoms that might be associated with multisystem disease; however, this would be reliant on the use of more specific Read codes within these databases. Finally, the use of data from primary care is only as useful as the information that is given to the GP by the patient and the data that are then recorded in the database. The date that symptoms are first reported to GPs will not equate to the date that patients first experienced the symptoms and it is likely that they will have only reported symptoms to their GP once they become problematic enough that self-management is no longer sufficient. When these data were presented to a group of patients from a Lupus UK support group, they unanimously reported not telling their GP about some of their symptoms as they were embarrassed and did not want to sound like a hypochondriac; a sentiment that is echoed in the qualitative literature on SLE diagnosis.^42^ Despite the limitations of the study in this respect, the data do represent the working clinical record for GPs in the UK and the potential for developing flagging software in the future should be considered.

In conclusion, despite awareness campaigns, the delay from symptom onset to SLE diagnosis is still long, especially in older-onset SLE. Musculoskeletal, mucocutaneous and neurological symptoms are the most frequent early symptoms of SLE and therefore SLE should be considered in patients presenting frequently or chronically with these symptoms, including patients over the age of 50 years.
